# MicroRNA-186 induces sensitivity of ovarian cancer cells to paclitaxel and cisplatin by targeting ABCB1

**DOI:** 10.1186/s13048-015-0207-6

**Published:** 2015-12-02

**Authors:** Kai-Xuan Sun, Jin-Wen Jiao, Shuo Chen, Bo-Liang Liu, Yang Zhao

**Affiliations:** Department of Gynecology, The First Affiliated Hospital of China Medical University, Shenyang, 110001 P.R. China; Department of Gynecology, The Affiliated Hospital of Qingdao University, Qingdao, 266003 P.R. China

**Keywords:** Ovarian cancer cells, MicroRNA 186, ABCB1, Paclitaxel, Cisplatin, Drug resistance

## Abstract

**Background:**

Recent studies have shown that microRNAs may regulate the *ABCB1* gene (ATP-binding cassette, sub-family B [MDR/TAP], member 1). Computational programs have predicted that the 3’-untranslated region (3’-UTR) of *ABCB1* contains a potential miRNA-binding site for miR-186. Here, we investigated the role of miR-186 in sensitizing ovarian cancer cells to paclitaxel and cisplatin.

**Results:**

Human ovarian carcinoma cell lines OVCAR3, A2780, A2780/DDP, and A2780/Taxol were exposed to paclitaxel or cisplatin with or without miR-186 transfection, and cell viability was determined by MTT assay. Reverse transcriptase-polymerase chain reaction (RT-PCR) and Western blot analysis were used to assess the MDR1, GST-π, and *MRP1* expression levels. Dual-luciferase reporter assay was used to reveal the correlation between miR-186 and *ABCB1*. Lower miR-186 while higher MDR1 and GST-π mRNA expression levels were found in the A2780/Taxol and A2780/DDP cells than in the A2780 cells. After miR-186 transfection, all the cell lines showed increased sensitivity to paclitaxel and cisplatin. MiR-186 transfection induced apoptosis while anti-miR-186 transfection reduced apoptosis. The dual-luciferase reporter assay verified that that miR-186 combined with the 3’-untranslated region (UTR) of *ABCB1*. MDR1 and GST-π mRNA and protein expression levels were downregulated after transfection with miR-186 but upregulated following anti-miR-186 transfection compared to the mock and negative control cancer cells; however, the MRP1 expression levels did not significantly differ among the groups.

**Conclusion:**

Our results are the first to demonstrate that miR-186 may sensitize ovarian cancer cell to paclitaxel and cisplatin by targeting ABCB1 and modulating the expression of GST-π.

**Electronic supplementary material:**

The online version of this article (doi:10.1186/s13048-015-0207-6) contains supplementary material, which is available to authorized users.

## Background

Epithelial ovarian cancer is the fifth leading cause of cancer death in women and the leading cause of death from gynecological cancer [1]. The 5-year survival rate for all stages of ovarian cancer has been estimated at 35–38 %. The primary treatment of ovarian cancer is surgical resection of visible tumors followed by adjuvant chemotherapy such as paclitaxel and cisplatin, which are the conventional anticancer drugs with long-term clinical applications for cancer treatment with specific applications in ovarian cancer. As numerous patients with ovarian cancer eventually relapse following resistance to paclitaxel or cisplatin therapy, it is vital to identify novel and more effective treatments for human EOC.

MicroRNAs (miRNA) are endogenous, noncoding RNAs that direct gene repression by inhibiting the mRNA stability or translation [2]. An increasing body of evidence suggests that aberrant microRNA expression enhances the development of drug resistance by interfering with the expression of target proteins that may be drug transporters, drug targets, or cell apoptosis- and cell-cycle-related components, resulting in cells with different degrees of sensitivity to chemotherapeutic drugs. Studies have showed that miRNAs such as miR-27a [3], miR-106a [4], miR-133a [5], miR-145 [6], miR-181b [7], miR-218 [8], and miR-326 [5] may be involved in the development of drug resistance by regulating relative gene expression. *ABCB1* encodes a multi-drug-resistance gene (*MDR1*), and is the most prominent member of the ABC transporter family, and it is the most thoroughly investigated member of this family [9]. It is in the complex network of microRNAs (miRNAs) and transcription factors affecting the transport of chemotherapeutic drugs such as cisplatin and paclitaxel; furthermore, it’s often observed to be upregulated in chemotherapy-resistant cancer cell lines; therefore, it has been suggested to contribute to the phenomenon of drug resistance [10].

Previous evidence has indicated that miR-186 overexpression can lead to reduced expression of twist family bHLH transcription factor 1 (Twist1) along with morphological, functional, and molecular changes consistent with mesenchymal-to-epithelial transition, G1 cell-cycle arrest, and enhanced cell apoptosis, rendering the cells more sensitive to cisplatin [11]. Our computational programs predicted that the 3’-untranslated region (3’-UTR) of ABCB1 contains a potential miRNA-binding site for miR-186. Therefore, we investigated the role of miR-186 in sensitizing ovarian cancer cells to chemotherapy.

## Methods

### Cell culture and transfection

As previously introduced [12], Ovarian carcinoma cell lines OVCAR3 and A2780 (serous cystic adenocarcinoma), Cisplatin-resistant A2780 (A2780/DDP), and paclitaxel-resistant A2780 (A2780/Taxol) were maintained in RPMI-1640 (A2780/DDP, A2780/Taxol, and OVCAR3) or Dulbecco’s modified Eagle’s medium (DMEM; for A2780 cells) medium supplemented with 10 % fetal bovine serum (FBS), 100 units · mL^−1^ penicillin and 100 μg · mL^−1^ streptomycin. The cell lines were placed in humidified atmosphere of 5 % CO_2_ at 37 °C with or without paclitaxel or cisplatin treatment and miR-186 transfection using Lipofectamine-2000 in accordance with the manufacturer’s guidelines (Invitrogen). Untreated cells were designated as the control group.

### Cell viability assay

Cell viability was determined using the 3-(4,5)-dimethylthiahiazo (−z-y1)-3,5-di-phenytetrazoliumromide (MTT, Beyotime, Jiangsu, China) assay. Briefly, 2.5 × 10^3^ cells/well were seeded to the wells of a 96-well plate and allowed to adhere. At different time points, 20 μL of MTT solution was added to each well of the plate, and the plates were incubated for 4 h. Then, liquid was removed from the plate and 150 μL of DMSO was added to the wells, the mixture was agitated for 10 minutes, and the OD was measured at 490 nm.

### Dual-luciferase reporter assay

The *ABCB1* wild-type 3’-UTR target sequence was cloned into a luciferase vector containing the *Renilla* luciferase gene. Mutant 3’-UTR was also cloned. Human embryonic kidney (HEK)-293 T cells were cotransfected with miR-Mock or miR-ABCB1 mimics using Lipofectamine 2000 (Invitrogen). The cells were collected 48 h after transfection and analyzed using the dual-luciferase reporter assay system (Promega, Madison, WI), and the detected luciferase activity was normalized to the activity of *Renilla* luciferase. Each reporter plasmid was transfected at least three times, and each sample was assayed in triplicate. The wild sequence for ABCB1 (NM_000927) 3’ UTR: AACTTCTGCUUTAAAAAAGTTUUCUUUAAATATACCTACTCATTTTTGTGGGAATGG; while mutant sequence was AACTTCTGCGCTATGTGTGTCGUCUTGAAATATACCTACTCATTTTTGTGGGAATGG were designed and purchased from Shanghai Genechem Co.,Ltd (Shanghai, China).

### Real-time reverse transcriptase-polymerase chain reaction (RT-PCR)

Total RNA was extracted from the ovarian carcinoma cell lines using TRIzol® (Takara, Kyoto, Japan). Real-time RT-PCR was performed using 2 μg of total RNA using AMV reverse transcriptase and random primers (Takara, Kyoto, Japan). The PCR primers were designed according to the sequences in GenBank (Additional file 1: Table 1). cDNA amplification was performed according to the manufacturer’s protocol using an SYBR Premix Ex *Taq* II kit (Takara, Kyoto, Japan). All PCR experiments were accompanied with a no-template control and 18S as the internal control. The relative gene expression level (amount of target normalized to the endogenous control gene) was calculated using the comparative CT method: 2^–ΔΔCt^.

### Western blot analysis

Protein assays were performed according to the Bradford method using a Bio-Rad protein assay kit (Bio-Rad, Hercules, CA, USA). Denatured proteins were separated by sodium dodecyl sulfate-polyacrylamide gel electrophoresis (SDS-PAGE) on 8 % acrylamide gels, and then transferred to Hybond™ membranes (Amersham, Germany). The membranes were blocked overnight in 5 % skimmed milk in Tris-buffered saline with Tween®-20 (TBST). For immunoblotting, the membranes were incubated at 4 °C overnight with anti-MDR1 (Bioss, Peking, China) and anti-GST-π, anti-MRP1 (Proteintech Group, Chicago, USA) antibodies, rinsed with TBST, and incubated with anti-rabbit IgG antibodies conjugated to horseradish peroxidase (HRP; Dako, Carpinteria, CA, USA) at a dilution of 1:5000. After applying electrochemiluminescent (ECL)-Plus detection reagents (Santa Cruz, CA, USA), the protein bands were visualized using an X-ray film (Fujifilm, Tokyo, Japan). The immunoblots were washed with Western blotting stripping buffer (pH 2–3; Nacalai, Tokyo, Japan) and probed with monoclonal antibodies against GAPDH (1:2000; Proteintech Group, Chicago, USA).

### Statistical analysis

Statistical analyses were carried out using paired *t* test to compare the mean values among different groups. A *p* value of < 0.05 was considered statistically significant. SPSS 17.0 software (SPSS, Chicago, IL, USA) was employed to analyze all data.

## Results

MiR-186 overexpression sensitized ovarian cancer cells to paclitaxel and cisplatin

**Fig. 1:**
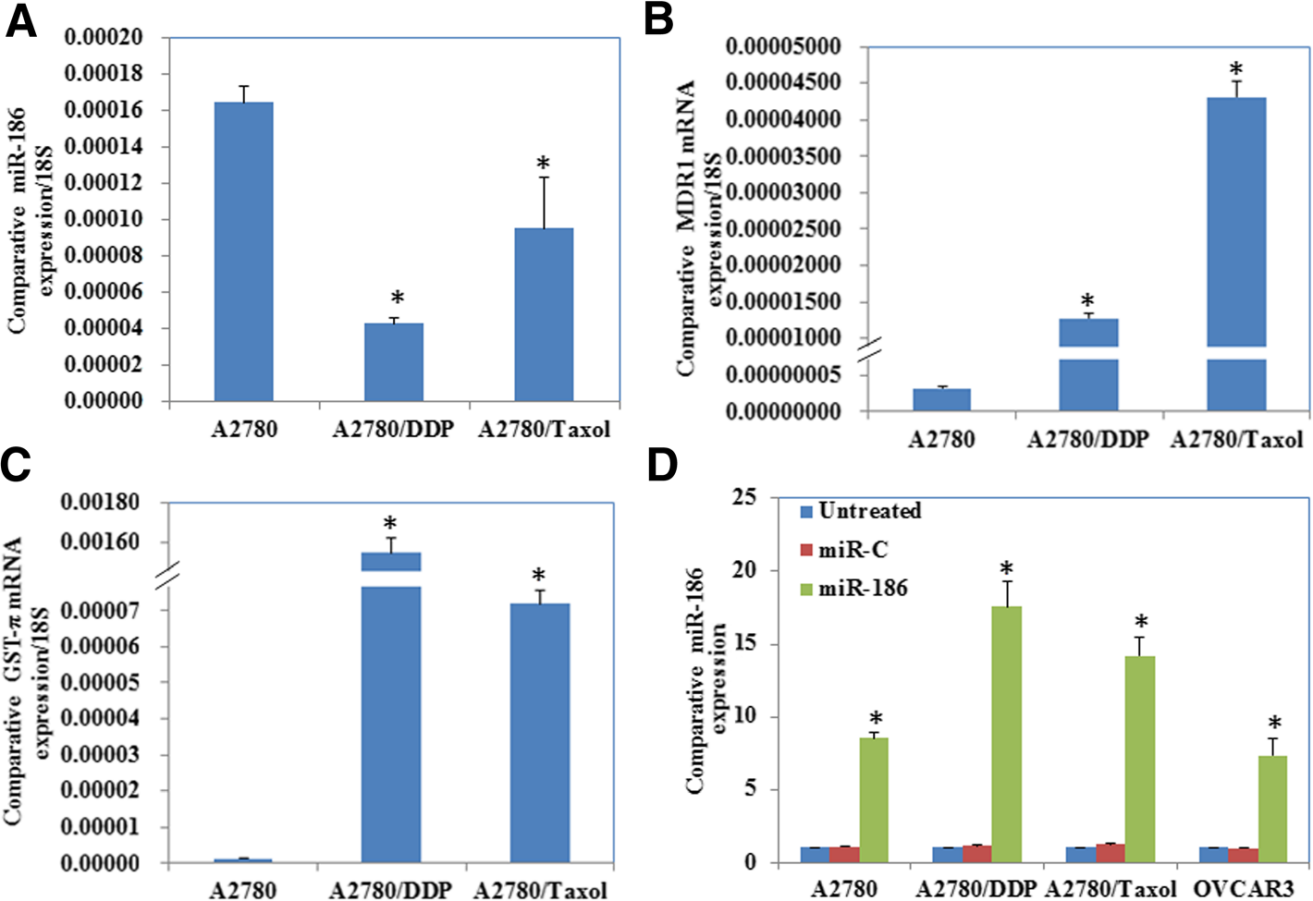
MiR-186, MDR1 and GST-π expression in ovarian cancer cell lines. Results of the RT-PCR showed (**a**) lower miR-186 expression level in A2780/DDP and A2780/Taxol than in A2780, (**b**) while higher MDR1 and (**c**) GST-π mRNA expression level in A2780/DDP and A2780/Taxol than in A2780. **d** MiR-186 transfection significantly induced miR-186 expression. Results are representative of three separate experiments. Data are expressed as the mean ± standard deviation. * *P* < 0.05

**Fig. 2:**
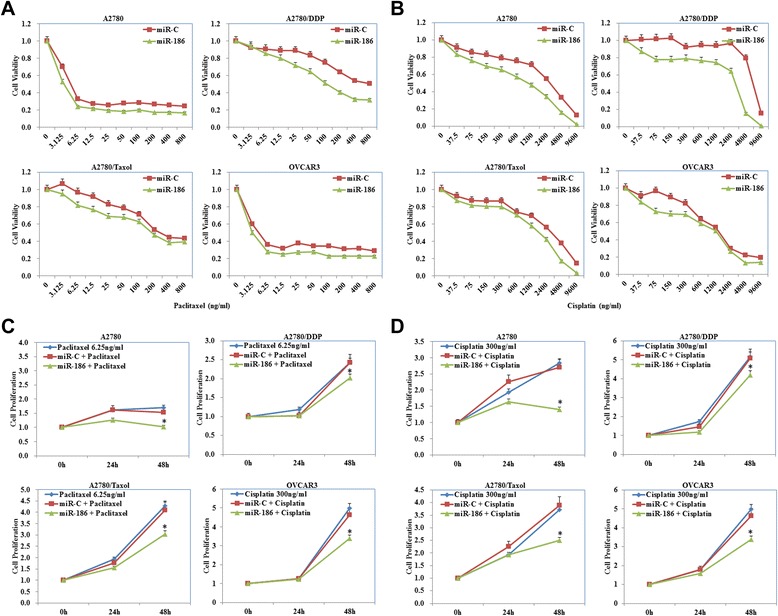
MiR-186 overexpression sensitized ovarian cancer cells to paclitaxel and cisplatin. **a** and **b** MiR-186 overexpression induced the sensitivity of ovarian cancer cells to paclitaxel and cisplatin compared with miR-C transfected cells. **c** and **d** Besides, miR-186 transfection also reduced cancer cell proliferation. Results are representative of three separate experiments. Data are expressed as the mean ± standard deviation. * *P* < 0.05

Results of the RT-PCR revealed lower miR-186 expression level in A2780/DDP and A2780/Taxol than in A2780 cells (Fig. 1a, *p* < 0.05), while higher MDR1 and GST-π mRNA expression level in A2780/DDP and A2780/Taxol than in A2780 cells (Fig. 1b & c, *p* < 0.05). MiR-186 overexpression (Fig. 1d, *p* < 0.05) induced the sensitivity of ovarian cancer cells to paclitaxelb (Fig. 2a) and cisplatin (Fig. 2b), compared with the untreated groups and miR-C transfected groups. Besides, miR-186 transfection also reduced cancer cell proliferation (Fig. 2c & d, *p* < 0.05).

### MiR-186 induces apoptosis

**Fig. 3:**
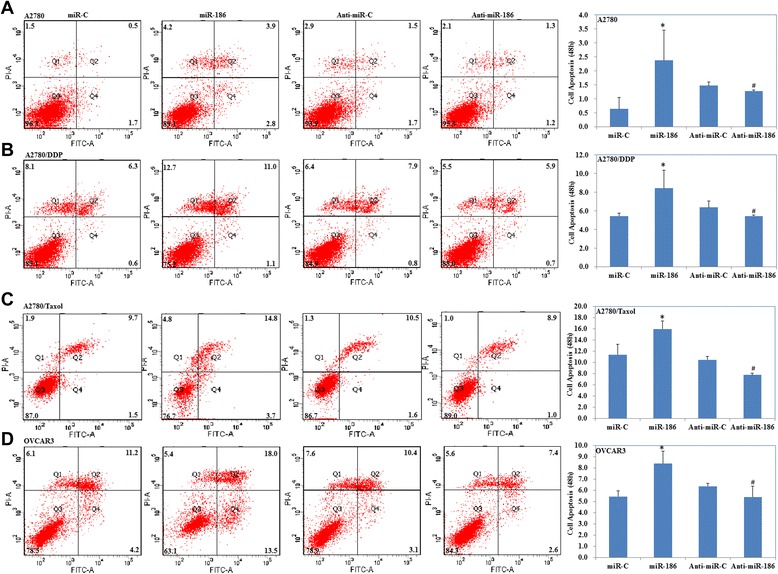
MiR-186 induces cell apoptosis. Transfection with miR-186 could induce ovarian cancer cell lines A2780 (**a**), A2780/DDP (**b**), A2780/Taxol (**c**) and OVCAR3 (**d**) cell apoptosis while anti-miR-186 transfection inhibited cell apoptosis in ovarian cancer cell lines. Results are representative of three separate experiments. Data are expressed as the mean ± standard deviation. * Compared with miR-C group; # Compared with anti-miR-C group, *P* < 0.05

We investigated the role of miR-186 on cell apoptosis. Our results showed that restoring miR-186 could induce apoptosis while transfection with anti-miR-186 inhibited apoptosis in ovarian cancer cell lines (Fig. 3, *p* < 0.05).

### Bioinformatics and luciferase reporter assay

**Fig. 4:**
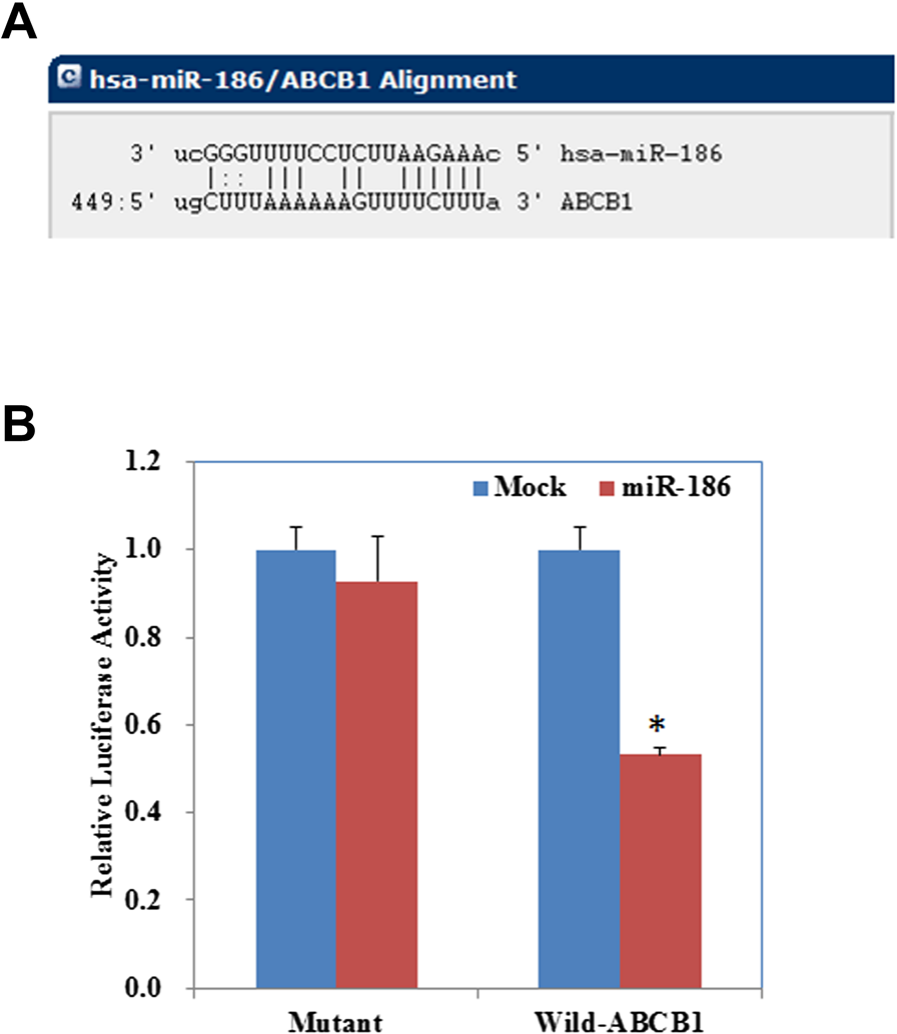
Bioinformatics and luciferase reporter assay results. **a** The 3’-untranslated region (3’-UTR) of *ABCB1* contains a potential miRNA-binding site for miR-186. **b** MiR-186 significantly decreased the relative luciferase activity of the wild-type ABCC1 3’UTR compared with the mutant ABCC1 3’UTR. Results are representative of three separate experiments. Data are expressed as the mean ± standard deviation. * *P* < 0.05

Computational programs predicted that the 3’-UTR of *ABCB1* contains a potential miRNA-binding site for miR-186 (Fig. 4a). We performed luciferase reporter assays with the wild-type or mutant 3’UTR of ABCB1. Our results demonstrate that miR-186 significantly decreased the relative luciferase activity of the wild-type ABCC1 3’UTR compared with the mutant ABCC1 3’UTR, indicating that miR-186 may directly bind to the 3’UTR of ABCC1 (Fig. 4b, *P* < 0.05).

### MiR-186 overexpression downregulates the expression of MDR1 and GST-π

**Fig. 5:**
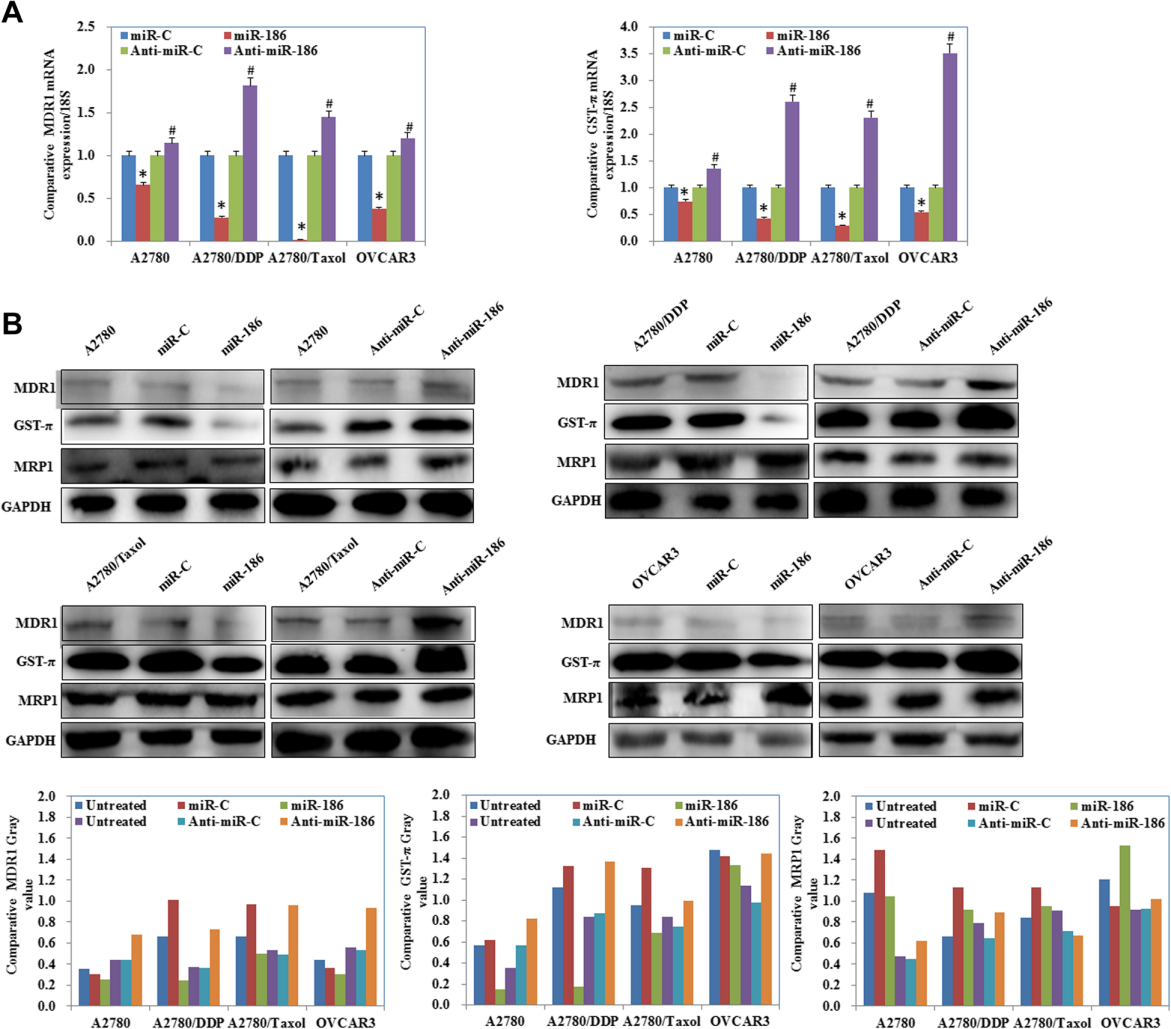
MiR-186 downregulates MDR1 and GST-π expression levels. **a** MRNA and (**b**) protein expression levels of MDR1 and GST-π with miR-186 andanti-miR-186 transfection. Results show that mir-186 decreased both mRNA and protein levels of MDR1 and GST-π while anti-miR-186 increased both these levels. However, the MRP1 expression levels did not significantly differ among the groups. Results are representative of three separate experiments. Data are expressed as the mean ± standard deviation. * Compared with miR-C group; # Compared with anti-miR-C group, *P* < 0.05

MiR-186 overexpression inhibited the expression levels of MDR1 and GST-π mRNA in ovarian cancer cell lines while anti-miR-186 transfection upregulated MDR1 and GST-π expression compared with negative control cells or mock transfected cells (Fig. 5a, *p* < 0.05). Similarly, results of the Western blot analysis revealed that the MDR1 and GST-π protein levels were decreased after miR-186 transfection but remained higher than the corresponding levels in the negative control and mock transfected groups (Fig. 5b), however, the MRP1 expression levels did not significantly differ among the groups.

## Discussion

MiRNAs frequently target 3’-UTRs and participate in numerous cellular events such as proliferation, differentiation, and apoptosis [13–15]. Studies have shown that miRNAs play an important role in modulating the sensitivity of cancer cells to chemotherapeutic agents [16, 17]. Thus, microRNAs can be promising diagnostic and prognostic molecular biomarkers as well as therapeutic targets in cancers [18, 19].

MiR-186 has been reported to regulate glycolysis through *Glut1* during the formation of cancer-associated fibroblasts [20]. Cui et al. reported that miR-186 targets *ROCK1* to suppress the growth and metastasis of non-small cell lung cancer cells [21]. Cai et al. reported that miR-186 downregulation correlates with poor survival in lung adenocarcinoma [22]. These studies suggest that miR-186 may function as a tumor suppressor gene. Our results showed that both A2780/DDP and A2780/Taxol cells expressed miR-186 at lower levels than A2780. MiR-186 overexpression increased the sensitivity of ovarian cancer cell lines to paclitaxel and cisplatin compared with the negative control or mock cells, miR-186 transfection induced cell apoptosis while anti-miR-186 transfection reduced cell apoptosis, suggesting that miR-186 may inhibit the development of drug resistance in ovarian cancer cells.

MDR (multi-drug resistance) is a major obstacle to the success of cancer chemotherapy, and it involves cancer stem cell regulation, ABC transporter family, miRNA regulation, hypoxia induction, DNA damage and repair, apoptosis induction, autophagy induction, and epigenetic regulation. The ABC transporter family is known to have 12 putative drug transporters [23, 24], including MDR1 (encoded by *ABCB1*) and MDR-associated protein-1 (MRP1, encoded by *ABCC1*) [25]. The predicted seed region in the 3’-UTR of *ABCB1* and *ABCC1* showed that both these genes are the direct targets of miR-186. However, our results showed that miR-186 overexpression downregulated both the mRNA and protein expression levels of MDR1 and GST-π in the ovarian cancer cell lines compared to the negative control cells or mock transfected cells, while there was no significant difference in the expression of MRP1. Therefore, we suggest that miR-186 may increase cell sensitivity of ovarian cancer cells lines to paclitaxel and cisplatin by targeting *ABCB1* but not *ABCC1*. Studies have demonstrated the importance of increased MDR1 expression in the development of MDR, as this glycoprotein can help cells develop drug resistance by pumping drugs out of the cells and decreasing the intracellular drug concentration [26–31]. Moreover, the π isoform of GST, which is a member of the GST family and has been shown to be responsible for the excessive intensity of detoxification of cytostatics, was shown to have functional polymorphisms that could potentially affect the metabolism of chemotherapeutic agents and influence the efficacy of chemotherapy and cancer survival [32]. Studies have shown that GST dysfunction may improve ovarian cancer survival after postoperative chemotherapy; evaluation of the functional polymorphisms of GST may help arrive at a prognosis of ovarian cancer prognosis [33, 34]. Based on these findings and our study results, we consider that miR-186 may inhibit the development of drug resistance by targeting *ABCB1* and regulating GST-π expression in ovarian cancer cells. Importantly, we find that combination of miR-186 with chemotherapeutic agents can increase the sensitivity of ovarian cancer cells to paclitaxel.

Ours is the first study to demonstrate that miR-186 overexpression may increase the sensitivity of ovarian cancer cells to paclitaxel by targeting ABCB1 and modulating GST-π. Further studies are required to determine the molecular mechanisms and its clinical manipulation in the future.

## Conclusions

In conclusion, we demonstrated for the first time that miR-186 overexpression may increase the sensitivity of ovarian cancer cells to paclitaxel and cisplatin by targeting ABCB1 and modulating GST-π. Further research about the MDR-related cancer therapy will determine the contribution of certain mechanisms to the resistance of chemotherapeutics.

## Additional file

Additional file 1: Table 1. Primers for RT-PCR (DOC 31 kb)
