# Supplementary material for: MicroRNA-186 induces sensitivity of ovarian cancer cells to paclitaxel and cisplatin by targeting ABCB1
Source: J Ovarian Res. 2015 Dec 2;8:80. doi: 10.1186/s13048-015-0207-6 (PMC4667519; doi:10.1186/s13048-015-0207-6)
Supplement: Additional file 1: Table 1. — Primers for RT-PCR (DOC 31 kb) [file 13048_2015_207_MOESM1_ESM.doc]

**Supplementary Table 1:** Primers for RT-PCR

| **Gene** | **Primer sequence** | **Target sequence** | **AT**  **(^o^C)** | **Product size (bp)** | **Extension time (sec)** |
| --- | --- | --- | --- | --- | --- |
| *GST-π* | F: 5'-TTTCGCCGCCGCAGTCT-3'  R: 5'-TCCACGGTCACCACCTCCTC-3' | NM_000852  225-359 | 60 | 135 | 34 |
| *MDR1* | F: 5'-AGGCTATCATTACTCTTTACC-3'  R: 5'-TCTGGCTTCCGTTGC-3' | NM_000927  24-184 | 60 | 161 | 34 |

AT = annealing temperature
